# Editorial: Non-clinical Approaches to Improve Outcomes in Persons With Mental Disorders

**DOI:** 10.3389/fsoc.2022.967508

**Published:** 2022-07-05

**Authors:** Sharon Lawn, Helen Brooks, Keith Sutton, Emily Vicary, Anton N. Isaacs

**Affiliations:** ^1^College of Medicine and Public Health, Flinders University, Adelaide, SA, Australia; ^2^Division of Nursing, Midwifery and Social Work, Faculty of Biology, Medicine and Health, University of Manchester, Manchester, United Kingdom; ^3^Monash Rural Health, Monash University, Clayton, VIC, Australia

**Keywords:** non-clinical approaches, severe and persistent mental illness, personal recovery, holistic approach, music therapy, employment, Individual Placement and Support (IPS), peer support

Biomedical approaches such as medication and health professional delivered therapies are considered the mainstay of treatment for people considered to have severe or enduring mental ill-health. They have dominated the mental health discourse and research for many decades (Corrigan and Penn, 1997; Lebowitz and Appelbaum, 2019) with little or no focus on social determinant-informed interventions in the mental health research literature. Randomized controlled trials, many funded by pharmaceutical companies, have been the dominant source of evidence in this field. They have largely determined what processes and outcomes are important to measure and also how they are measured. Whilst their contribution is important, they may not be the best way to evaluate complex mental health interventions in relation to individuals' holistic recovery needs and experiences (Greenhalgh and Papoutsi, 2018; Paparini et al., 2020).

Biomedical approaches can ameliorate symptoms, prevent relapse, and help individuals to manage stress and understand how to self-care for the clinical recovery from mental illness (National Institute for Health and Care Excellence, 2014). However, much more is needed to address issues such as stigma, marginalization, and citizenship in the community for people with mental ill-health. Arising from significant lived experience advocacy over many decades, personal recovery is increasingly seen as part of good mental health care (Slade, 2009). Consequently, calls for lived experience perspectives are pervasive in the policies and practice guidelines of mental health service systems in many countries. Personal recovery relates to “a way of living a satisfying, hopeful, and contributing life even with limitations caused by the illness” (Anthony, 1993, p.11). Leamy et al. (2011), as one example of this shift in thinking, propose that personal recovery is built on the five processes of connectedness, hope and optimism, identity, meaning and purpose, and empowerment.

The dominance of clinical approaches has meant that the evidence for non-clinical approaches has lagged behind, even though existing treatments continue to have limited success in improving long-term clinical and personal recovery outcomes for many individuals, particularly for people with a diagnosis of schizophrenia (McBain et al., 2020). In short, there is a need to broaden the evidence base to investigate approaches that may contribute to a person's holistic recovery needs and experiences.

Therefore, this special issue focuses on a variety of non-clinical models and approaches to care in an effort to help build that evidence base. The studies exemplify the broad diversity of approaches that make up more holistic approaches to care. They demonstrate a variety of different psychosocial needs that contribute to mental health recovery and are drawn from research in the United Kingdom (Krishna Priya et al.), Australia (McDowell et al.), the United States (Chen et al.), and Slovenia (Makivić et al.).

Krishna Priya et al. used a mixed methods approach to investigate the impact of music for 41 people with a diagnosis of anorexia nervosa, also asking them their views on the potential therapeutic uses of music in their lives, the management of their mental health, and their recovery. They found that the impacts were predominantly positive. Of interest, most participants reported that listening to music evokes varying emotions in them (83%) which could be of a positive or negative nature. Music served an important role in both positive and negative memories, which is important when considering the links between mental illness and trauma for many individuals. Also of note, most participants reported that music helps to distract them (85%), helps with loneliness (59%) and helps them feel more connected to others (58%). They emphasized that music could be beneficial and important aspects of anorexia nervosa care, particularly for helping them to deal with and regulate their emotions and ameliorate loneliness and relationship difficulties. Many participants expressed the desire to attend music therapy to actively learn about and incorporate it into their management of their anorexia nervosa. Many of these benefits resonate with the elements of personal recovery noted above. They also align with a psychotherapeutic perspective, and the authors conclude that even negative emotions such as anger elicited by music could be therapeutically helpful, particularly for individuals with eating disorders who may have difficulty with expressing these negative emotions. Music may help them to recognize, regulate, and express these difficult emotions (Mallorquí-Bagué et al., 2020), helping both the person and their healthcare provider to access negative emotions and work through processing them.

In a narrative review of vocational service models to improve job tenure, McDowell et al. remind us of the importance of employment as a valued social role, a core social determinant of health and a right of citizenship for people with severe and enduring mental illness. The sense of meaning, identity, and belonging gained from employment is clearly aligned with personal recovery. This review focuses specifically on Individual Placement and Support (IPS) which is an evidence-based model of employment support known to improve job attainment and job retention for this population and address the challenges they are known to face. The review also found evidence for several adjunct interventions that, when combined with IPS, enhance job retention. These interventions include skills training, cognitive interventions, psychological interventions, and supported education, as well as more creative approaches such as social firms. They emphasize the important role of peer support and support from family and friends and the important, emerging evidence for how employment specialist practices, technology, self-management, and workplace accommodations can each play a positive role in influencing job tenure. The authors conclude that service providers would benefit from a greater focus on non-clinical vocational approaches to improve the employment experiences of this population. They argue that a recovery focus is unlikely to fully meet the diversity of needs if it does not also consider the context (Slade and Longden, 2015). They therefore call for a shift in focus from challenges being seen solely as situated within the individual to a greater focus on the systems and structures that impact on job access and tenure for this population.

Loneliness and social connection are consistently rated as significant issues for personal recovery in the mental health field. Chen et al.'s study involves a creative use of technology to measure, in real-time, social ambiance and its potential for enabling effective assessment and interventions for social isolation for people with depressive and psychotic illnesses. They argue that existing research is largely focused on determining loneliness, rather than isolation, and lacks focus on the rich social environment in which the individual is located and interacts. The authors describe their development of an automated, objective and privacy-preserving Social Ambiance Measure (SAM) that converts audio recordings collected from wrist-worn audio-bands. The four ambiance levels (from none to active) signifying overall social activity in the environment. They tested the SAM with a small sample 18 participants with either depression or psychosis and with 13 matched controls without mental health diagnoses. They found that social ambiance patterns and time spent at each ambiance level differ between these groups. Individuals with depression/psychosis spent less time in diverse environments and in environments with more active ambiance levels. The authors propose that objectively measuring social ambiance patterns can be used as a marker of sociability, improve our understanding of social isolation and its importance for people with mental ill-health, and can inform the development of more effective interventions to assist individuals to build the quality of their efforts to be sociable and connected in within their networks and community, and improve mental health outcomes.

Makivić et al. undertook a Delphi study with decision-makers, mental health service experts, and consumers of mental health services, looking at mental health needs assessment during the COVID-19 pandemic. The collaboration between these stakeholders was particularly noted as a strength of the study. The research was driven by concern for the needs of people with pre-existing mental health diagnoses (Li et al., 2020), the widespread psychological distress reported in many populations, and the potential for long-term increases in the number and severity of mental health problems during the pandemic (United Nations, 2020). The results indicated the importance of reorganizing services in the direction of improving local accessibility and strengthening services network to build their capacity for immediate responses to the psychological, health, and social needs of individuals, particularly those arising from crisis situations. In Slovenia, the study's results have already informed the formulation of the Action Plan of the National Mental Health Program and its implementation.

## Author Contributions

SL wrote the first drafts. HB, KS, EV, and AI reviewed the drafts. All authors approved the final version of the manuscript.

## Conflict of Interest

The authors declare that the research was conducted in the absence of any commercial or financial relationships that could be construed as a potential conflict of interest.

## Publisher's Note

All claims expressed in this article are solely those of the authors and do not necessarily represent those of their affiliated organizations, or those of the publisher, the editors and the reviewers. Any product that may be evaluated in this article, or claim that may be made by its manufacturer, is not guaranteed or endorsed by the publisher.
